# Hydrodynamic cavitation for lignocellulosic biomass pretreatment: a review of recent developments and future perspectives

**DOI:** 10.1186/s40643-022-00499-2

**Published:** 2022-01-25

**Authors:** Thiago Averaldo Bimestre, José Antonio Mantovani Júnior, Eliana Vieira Canettieri, Celso Eduardo Tuna

**Affiliations:** 1 Chemistry and Energy Department, School of Engineering, São Paulo State University UNESP, Guaratinguetá, SP 12516-410 Brazil; 2 Center for Weather Forecasting and Climate Studies, National Institute for Space Research CPTEC/INPE, Cachoeira Paulista, SP 12630-000 Brazil

**Keywords:** Hydrodynamic cavitation, Lignocellulosic biomass, Pretreatment, Hydrodynamic cavitation reactor, Biorefinery

## Abstract

The hydrodynamic cavitation comes out as a promising route to lignocellulosic biomass pretreatment releasing huge amounts of energy and inducing physical and chemical transformations, which favor lignin–carbohydrate matrix disruption. The hydrodynamic cavitation process combined with other pretreatment processes has shown an attractive alternative with high pretreatment efficiency, low energy consumption, and easy setup for large-scale applications compared to conventional pretreatment methods. This present review includes an overview of this promising technology and a detailed discussion on the process of parameters that affect the phenomena and future perspectives of development of this area.

## Introduction

With high population growth rates, the global population should reach 9.7 billion by 2050 with estimated energy consumption of 851 quadrillion BTU in 2040 (Bhowmick et al. 2018). Associated with quick global economic development arises with the concern of power supply in the next decades and with the increased demand for fuel and chemicals. With the strong dependency of the international power system for fossil fuels and the preoccupation of sources depletion, global climate changes and negative impact on the people's health due these sources are clear the necessity of alternative energy resources, sustainable with clean and renewable raw materials (Mahmood et al. 2019). In this sense, extensive research has been done to develop biotechnological routes of low environmental impacts that use lignocellulosic biomass residual abundantly generated throughout in the agricultural and forestry sectors to bioenergy, biofuel, and bioproducts production (Madison et al. 2017). However, due to the complex structure and recalcitrance of lignocellulosic biomass, a pretreatment step is required to make its use viable.

In this path, hydrodynamic cavitation arises as a promising technological route to lignocellulosic biomass pretreatment (Wu et al. 2019). Hydrodynamic cavitation phenomena occur through mechanical constrictions as Venturi pipes, orifice plates, and throttling valve, which cause a sufficient pressure change to form vapor microcavities that collapse releasing high energy amount, inducing physical and chemical transformations, which favor the lignin–carbohydrate matrix disruption (Bimestre et al. 2020). Although the use of hydrodynamic cavitation as a pretreatment of lignocellulosic biomass has been studied for some time, the number of published articles is relatively small (less than 35) compared to other emerging pretreatment methods, indicating that HC did not attract much attention of researchers (Sun et al. 2021a).

This review discusses the recent developments over hydrodynamic cavitation for the lignocellulosic biomass waste pretreatment, beyond the influence parameters of these phenomena and perspectives of future development on this area.

## Lignocellulosic biomass and pretreatment step

Lignocellulosic biomass is grown as an energetic culture and does not compete with food cultures (Ji et al. 2020).

The lignocellulosic waste is commonly divided into two classes: residues abandoned in the field after the harvest and residues detached from industrial procurement. Usually, part of the lignocellulosic waste is destined to boilers in the power production at agricultural industries, however, has a large available surplus, which if not properly disposed of can bring significant environmental problems (Zuin et al. 2018). Thus, lignocellulosic biomass conversion provides, besides a renewable energy source, offers a reduction of excessive waste accumulation during processing. The generation of lignocellulosic waste in the world is estimated to be over 13 billion tons per year (Arias et al. 2021), which would be readily available to produce a range of higher value-added products as biofuels, power, and chemicals, reinforcing the biorefinery concept (Hassan et al. 2018).

The lignocellulosic biomass is composed of cellulose, hemicellulose, and lignin in addition to extractives in smaller proportions. One of the principal compounds of the vegetable biomass cell wall is cellulose, which is a linear polysaccharide with repetitive units named cellobiose (disaccharide d-glucose) that are joined by $\beta - \left( {1 \to 4} \right)$ bonds. Strong intramolecular or intermolecular hydrogen bonds occur through free hydroxyl OH groups of cellulose molecules. The intramolecular bond occurs between hydroxyl groups of the same molecule, giving it a certain stiffness. The intermolecular bond on the other hand occurs between hydroxyl groups of adjacent chains responsible for fibril formation and ordered structures, which are accountable for generating cellulose fibers (Sharma et al. 2019). The cellulose molecules are extremely solid and low reactive, with a high degree of polymerization and cristanility (polymerization degree of 500–15,000) having crystalline and amorphous regions; so that for cellulose solubilization degradation of fibril structures is necessary, breaking intermolecular bonds and obtaining glucose as a product (Kleingesinds et al. 2018).

Hemicellulose is a random heterogeneous and branched polymer, composed of different polysaccharides (poliosis) and includes pentoses (xylose and arabinose), hexoses (glucose, galactose, and mannose), and uronic acids. The branched nature of hemicellulose allows the formation of strong bonds with cellulose (hydrogen bonds) and lignin (covalent bonds) that increase the lignocellulosic material stiffness. Furthermore, hemicellulose has different sugar units that are linked together, does not form a fibrous arrangement as cellulose, and has amorphous regions. The hemicellulose has a low polymerization degree (DP 50–200) (Koupaie et al. 2019). Different types of hemicellulose can be found in nature, which is composed of different polymers, e.g., xyloglucans, xylans, and mannanas, of which xylan is more abundant. The xylans can be classified in homoxylans, arabinoxylans, glucuronoxylan, and arabinose glucuronoxylan, the last one is the main component of agricultural residue (Banerjee et al. 2019). Lignin is a big and complex compound, made of phenylpropane units that are bonded in a three-dimensional structure.

The main lignin monomers are the cumarylic alcohol (p-Hydroxyphenyl), the coniferyl, and synapse alcohols (Renault et al. 2019). Lignin acts as a “glue” that joins cellulose and hemicellulose to form a three-dimensional stiff structure cell wall of plants. These features turn lignin into the most resistant lignocellulosic biomass component to chemical and biological degradation (Zheng et al. 2014). If the lignin content is high, higher is the degradation resistance (Li et al. 2018).

Due to the complex structure of lignocellulosic biomass, its use without a pretreatment step results in lower sugar yields in biorefineries. Hence the complex lignocellulosic matrix’s highly recalcitrant deconstruction presents itself as the main challenge to be surpassed through the pretreatment step. The pretreatment step aims to open the recalcitrant structure of lignocellulosic material, providing an easier action of enzymes at a later stage of enzymatic hydrolysis, favoring the recovery of monomer sugars that are present in the carbohydrate fractions for later use in bioprocesses (Luo et al. 2021; Verdini et al. 2021). A successful pretreatment step should promote the biomass delignification, modifying and/or removing hemicellulose, decrease the crystallinity degree of cellulose, and increase the surface area and porosity, thus increasing the digestibility extension. In addition, it should limit the production of inhibitors, reduce production costs and the energy demand (Kumari and Singh 2018; Ponnusamy et al. 2019; Rezania et al. 2020; Zheng et al. 2014; Sun et al. 2021a).

The pretreatment of lignocellulosic material can be classified as physical, chemical, physical–chemical, biological, or a combination of these, which will depend on the required separation degree and of the proposed end (Lee and Park 2020) (Kumar and Sharma 2017). Through the physical pretreatment methods can highlight milling, extrusion, freezing, and microwave irradiation. These methods decrease the particle size and increase the superficial lignocellulosic material area but are not effective in isolation and are employed combined (Kumari and Singh 2018). Among the chemical pretreatment methods can highlight organosolv, ozone, and ionic liquids methods. The acid and alkaline methods are the most extensively employed due to providing high cellulose and hemicellulose solubilization and lignin removal.

Acid pretreatment methods are performed at low concentrations with high temperatures; furthermore, specific equipment must work in severe chemical conditions to avoid reactor corrosion. Differently, the alkaline pretreatment can be performed with low temperatures with long residence time, although have high water consumption to wash the pretreated biomass, which is not environmentally friendly (Haldar and Purkait 2021). The physical–chemical pretreatments are ammonia fiber explosion (AFEx), autohydrolysis or steam explosion pretreatment, liquid hot water (LWG), wet oxidation (WO), and ultrasonication (US) (the US is a physical pretreatment used for pretreating lignocellulosic biomass for their conversion to bioproducts but when the US is used with an acid or base it can be called a physicochemical pretreatment) (Abraham et al. 2020). The biological methods most employed are fungal (Giri and Sharma 2020) microbial, and enzymatic (Rai et al. 2019). Detailed information on emerging technologies for pretreating of lignocellulosic biomass can be found in many reviews such as (Yiin et al. 2021; Mankar et al. 2021; Kumar et al. 2020; Sarker et al. 2021).

The generation of hazardous environmental waste and/or high energetic inputs are the bottleneck of lignocellulosic biomass pretreatment processes and there is an urgent need for green technological solutions for this challenge (Ong et al. 2021).

## Hydrodynamic cavitation

The cavitation phenomena occur when vapor microbubbles are formed in a liquid flow, grow and then collapse due to sudden reductions of local pressure. Based on generation modes the cavitation is classified into four types: acoustic, hydrodynamic, optic, and particle. Meanwhile, only acoustic and hydrodynamic cavitation was efficiently considered in physical–chemical change production which is desired in pretreatment processes (Thanekar and Gogate 2019; Li et al. 2020). The acoustic cavitation is obtained by propagating an ultrasonic signal (20–100 kHz) of high amplitude in a liquid being the most frequent way to produce cavitation on a laboratory scale (Hilares et al. 2017a).

**Fig. 1:**
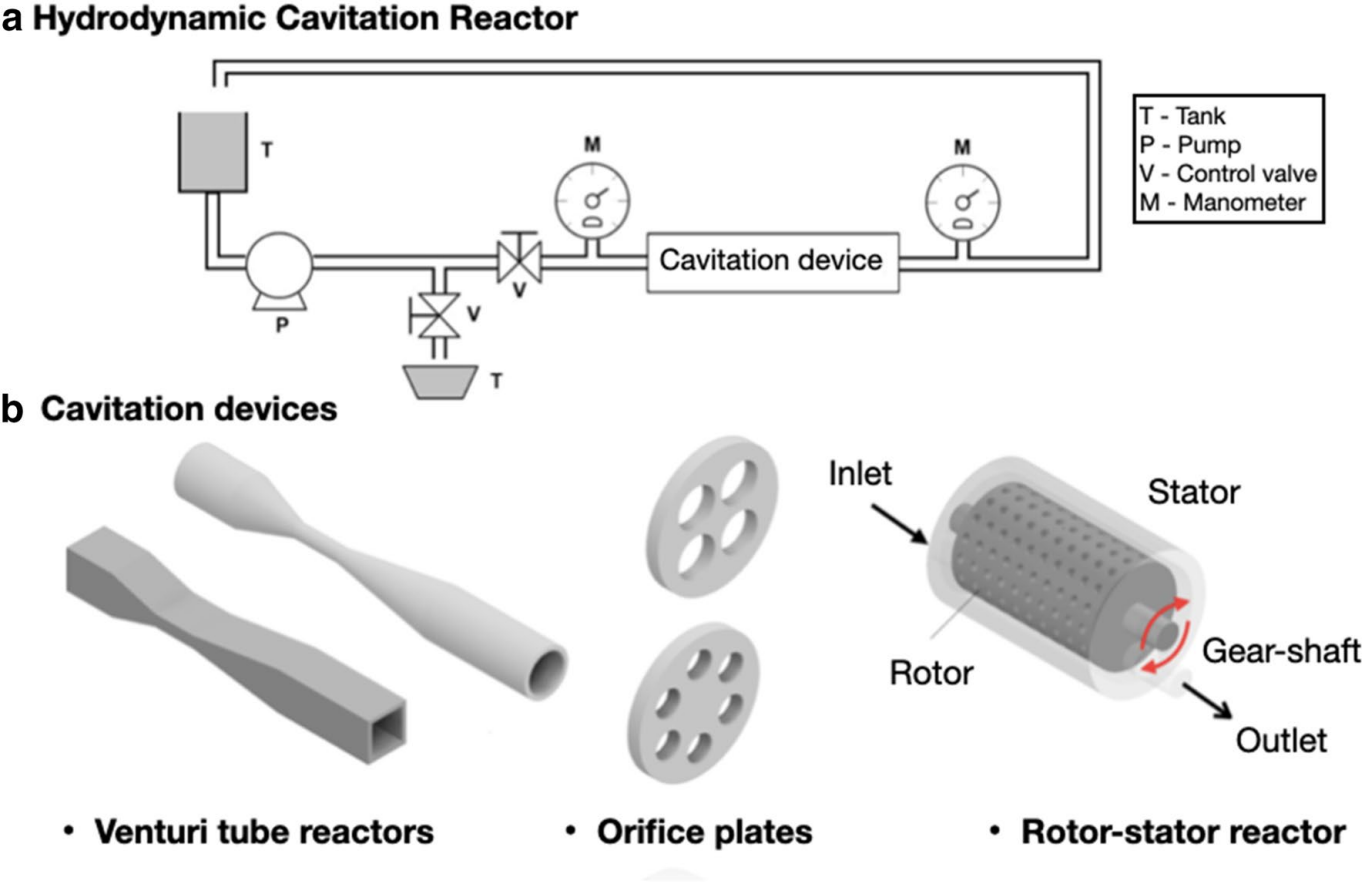
Schematic diagram of different HC systems

Hydrodynamic cavitation is more efficient than acoustic cavitation in many applications due to its capacity to oxide organic substances allied to low-cost operation, easy scalability, high power efficiency, and less polluting with no byproducts formed (Raut-Jadhav et al. 2016; Nakashima et al. 2016). Figure 1 shows a hydrodynamic cavitation reactor and its components as well as some cavitation device options.

It is produced through mechanical constrictions, such as Venturi pipes, orifice plates, and throttling valves. The cavitation can be explained based on the velocity–pressure fluid relationship in agreement with Bernoulli’s equation. When fluid flows through the constriction the pressure falls below the liquid–vapor pressure with flow temperature, then form vapor cavities, which collapse in the downstream region and create highly destructive shockwaves with huge pressures, vigorous turbulence, and generate strain (Badve et al. 2013). This collapse is sufficiently strong to release large energy amounts in a short space (Shrikant and Khambete 2017). The vapor cavity implosion can locally generate high temperatures of 5000–10,000 K and pressures of 1000–2000 atm, which induces physical and chemical transformations, producing strong oxidative radicals such as the hydroxyl radical (OH-) due to water molecules decomposition and organic molecules decomposition/pyrolysis trapped inside or nearby vapor cavities contributing to structural disintegration and biomass porosity increase (Kim et al. 2015). Concerning vapor bubbles, dynamic exists two main features: the maximum size bubble and its traveled distance before the collapse, i.e., its useful life. After vapor bubbles form, an expansion process begins due to quick liquid vaporization. If during the expansion process the vapor bubbles are submitted to a pressure higher than vapor pressure, its development is interrupted, the interior bubble pressure increases, and vapor condensation initiate resulting in bubble collapse. As the specific vapor volume is greater than the specific liquid volume the collapse will create void provoking shockwaves. The maximum bubble size defines the cavitation intensity. Bubbles grow under low pressure or high temperature, and larger bubbles implode with greater intensity and can generate more effects on a substance than smaller bubbles (Madison et al. 2017). Hydrodynamic cavitation can also be produced by an object mechanical rotating through a liquid, occurring in the centrifugal pump inlet and hydraulic turbine rotor outlet. Although it is an undesired phenomenon in hydraulic machinery areas, hydrodynamic cavitation is being applied in water and effluent treatment (Abramov et al. 2021; Sun et al. 2021a; Wang et al. 2020), biogas production (Zielinski et al. 2019; Saxena et al. 2019; Patil et al. 2016), cell disruption (Mevada et al. 2019), biodiesel production (Samani et al. 2021; Chipurici et al. 2019; Chitsaz et al. 2018), microalgae oil extraction (Waghmare et al. 2019; Lee et al. 2019), chemical reactors (Dhanke and Wagh 2020) and lignocellulosic biomass pretreatment (Thangavelu et al. 2018; Hilares et al. 2019).

### Hydrodynamic cavitation reactors

Hydrodynamic cavitation reactors are divided into two categories: non-rotating reactors, such as orifice plates or Venturi pipes and rotating reactors, where cavitation is generated on a region swept by high-velocity propellers (Sun et al. 2018a). Hydrodynamic cavitation reactor can also be categorized based upon its operating ways, as a pulsating hydrodynamic cavitation reactor (operating the reactor in cycles); continuous hydrodynamic cavitation reactor, and a shear-induced hydrodynamic cavitation reactor (Panda et al. 2020).

#### Non-rotational hydrodynamic cavitation reactors (NRHCRs)

The NRHCRs consist of a feed tank, a pump that makes circular mixture reactions through the reactor (cavitation chamber) as well as control valves, pressure transducers, and temperature gauge.

The Venturi pipe is composed of three sequential parts: a convergent section (nozzle), a throat, and a divergent section (diffusor). It can present circular or polygonal cross sections depending on the application and is historically applied to measure and to control the flow rate inflows, and recently is being employed in gas purification systems (Bal et al. 2019) solid–gas injectors (Jensen et al. 2018), jet hydraulic pumping (Ji et al. 2015) and hydrodynamic cavitation reactors (Simpson and Ranade 2019).

Orifice plates are simple, robust, relatively easy, and low cost to manufacture, instruments compared with venturi pipes. In an orifice plate reactor, the fluid flow follows through one or more constrictions in such a way that with a flow area decrease occurs a sudden velocity increase leads to pressures below liquid vapor pressure resulting in cavitation. When fluid flow passes by the orifice a flow reconfiguration occurs, which is named vena *contracta*. By that of Li et al. (2019), the Venturi pipes outperform orifice plates in disinfection applications with excellent performance in bacterial clusters generating denser cavitation and with more vapor bubbles formed. Moreover, Venturi pipes present higher flow rates than orifice plates to the same inlet energy leading to a greater treatment capacity and energetic efficiency, despite the higher manufacturing cost. Besides, in Venturi pipe systems with high solid load can be needed the use of special pumps as the diaphragm and helicoidal pumps with relatively higher costs than usual centrifugal pumps. For Saharan (2016), the pressure recovers smoothly in Venturi pipes due to the divergence angle leaving vapor cavities with enough time to grow to maximum size, increasing collapse intensity and the yield of cavitation, which does not occur in orifice plates. In the design of hydrodynamic cavitation, reactor is needed to consider some vital parameters in the process and the influence comprehension of these parameters in involved physical and chemical mechanisms. An important and widely used parameter to evaluate and optimize the hydrodynamic cavitation reactor performance is a dimensionless quantity known as cavitation number given as:

1 $$\sigma_{c} = \frac{{P_{2} - P_{\upsilon } }}{{0.5\rho U^{2} }}$$

where $P_{2}$ is the recovered pressure downstream of the cavitation device (Pa), *P*_v_ is the vapor pressure of the liquid at the operating temperature (Pa), $\rho$ is the density of the solution (kg/m^3^) and $U$ is the velocity at the constriction (m/s). The critical cavitation number corresponds to $\sigma_{c} = 1$, i.e., the number, where cavitation initiates. Under ideal conditions, cavitation occurs when $\sigma_{c} < 1$. Minimum cavitation number values result in more bubble generation and consequently increase the phenomenon intensity (Ijiri et al. 2018). However, it can be observed that the cavitation number does not consider local fluid dynamics. Therefore, the cavitation number is not a suitable parameter to compare the geometrical efficacy of cavitation devices (Dastane et al. 2019). A parameter called Cavitation Efficacy Ratio (CER) can be used to fix this problem. The CER is defined as follows:

2 $${\text{CER}} = \frac{{P_{{{\text{collapse}}}} }}{{P_{1} - P_{2} }}$$

where $P_{{{\text{collapse}}}}$ is the generated pressure after cavity collapse (Pa), $P_{1}$ is the inlet pressure (Pa) and $P_{2}$ is the outlet pressure (Pa). Essentially, the CER is the maximum theoretical efficacy of a cavitation system, where the collapse pressure represents the maximum energy amount that can be released by a cavity in a determined flow field, and the denominator is the permanent pressure loss (dissipated energy) during the process. The CER values can be useful to estimate the physical effects extension of cavitation. A cavitation device comparison based on CER can be directly applied to select cavitation devices (Dastane et al. 2019). The collapse pressure is determined by empirical correlation proposed by Gogate and Pandit (2001), which is easy-to-use and valid across the entire parameters range that commonly are employed in hydrodynamic cavitation applications (initial cavity size of 0.01–0.1 mm, inlet pressure of 1–8 atm, orifice diameter of 1–10 mm and holes free area percentage of 1–20%). The final developed correlation to hydrodynamic cavitation is given by the following:

3 $$P_{{{\text{collapse}}}} = 7527.(A)^{ - 2.55} \left\{ {( P_{i} )^{2.46} ( r_{0} )^{ - 0.8} (d_{0} )^{2.37} } \right\}$$

where $P_{{{\text{collapse}}}}$ is the bubble collapse pressure (atm), $A$ is the holes free area percentage (%), $P_{i}$ is the inlet pressure (atm), $r_{0}$ is the initial cavities radius (mm) and $d_{0}$ is the cavitation device diameter (mm). The correlation is only an indication of collapse pressure magnitude in hydrodynamic cavitation reactor. Another important parameter to consider in hydrodynamic cavitation reactor design is the cavitational yield developed by Gogate and Pandit (2001) expressed as:

4 $${\text{Cavitational yield}} = K.( P_{{{\text{collapse}}}} )^{w}$$

where the constant $K$ and exponent $w$ rely on reactor geometry, operational parameters and reaction type, which is being realized.

Despite the wide use of non-rotational reactors in numerous applications due to their structural versatility and ease of use, their effectiveness was considered unsatisfactory as they present considerable pressure losses due to severely restricted flow (Šarc et al. 2018; Tasalagkas et al. 2021).

#### Advanced rotational hydrodynamic cavitation reactors (ARHCRs)

The rotational reactors are based on centrifugal pumps, which have a modified rotor and a stator added in their housing (Kumar and Pandit 1999). In this type of reactor, the cavitation phenomenon is generated by numerous cavitation generation units (CGUs) located on the rotor and stator. A geometric structure of the CGUs is fundamental for the generation of cavitation as well as the effectiveness and economic efficiency. Furthermore, the frequency of energy release is significantly higher compared to NRHCRs (Sun et al. 2020a). Rotational reactors have exhibited excellent performance compared to non-rotational reactors for delignification (Lauberte et al. 2021), water treatments (Gostiša et al. 2021), biodiesel production (Samani et al. 2021), and sludge disintegration (Kim et al. 2020).

Researches investigating ARHCRs by experimental flow visualization (Kosel et al. 2019; Petkovšek et al. 2013; Šarc et al. 2018), computational fluid dynamics (Badve et al. 2015), and the characteristic experiment (Sun et al. 2018b, 2020b, 2021b) were presented to understand the mechanisms and design criteria of ARHCRs.

The effects of cavitation generation units (CGUs) on the performance of ARHCRs must be studied using computational fluid dynamics evaluating the effects of shape, diameter, interaction distance, height, and inclination angle of a CGU on the amount of cavitation generation and energy consumption of a representative ARHCR (Sun et al. 2021c).

To further improve the performance and develop the design criteria of the ARHCRs, a multi-objective optimization in the ARHCR framework is required. Sun et al. (2021d) performed for the first time a multi-objective optimization combining genetic algorithm (GA) and CFD. The chosen objectives were to minimize energy consumption and maximize the generation of hydrodynamic cavitation (Sun et al. 2021d).

To evaluate the thermal performance of ARHCRs, two important parameters are proposed by Sun et al. (2020b): Heat generation rate (HGR) and thermal efficiency (TE). The HGR produced by the ARHCRs is defined as the following:

5 $${\text{HGR}} = \rho_{{{\text{out}}}} .Q_{{{\text{out}}}} .C_{p} .{\Delta }T$$

where HGR is heat generation rate in MJ/h, Q_out_ is the outlet flow rate, m^3^/h, C_p_ is the specific heat of fluid, $\Delta T = T_{out} - T_{in}$, in °C, and $\rho_{{{\text{out}}}}$ is the outlet fluid density in kg/m^3^, defined as:

6 $$\rho_{{{\text{out}}}} = 1000 - 0.0178 x\left| {T_{{{\text{out}}}} - 4} \right|^{1.7}$$

The thermal efficiency (TE) according to Sun et al. (2020b) is defined as:

7 $${\text{TE }} = \frac{{{\text{HGR}}}}{{3.6 x P_{s} }}$$

where *P*_s_ is the measured shaft power of the electric motor in *kW*.

The cavitation index, $\sigma_{c}$, is defined as:

8 $$\sigma_{c} = \frac{{P_{{{\text{out}}}} - P_{{\text{v}}} }}{{0.5\rho .\left( {V_{{{\text{in}}}} + V_{{{\text{tangential}}}} } \right)^{2} }}$$

where *P*_out_ and *P*_v_ are the outlet static and saturated vapor pressures in Pa, respectively, *V*_in_ is the inlet velocity in m/s and *V*_tangential_ is the tangential velocity at the side of the rotor and can be calculated by:

9 $$V_{tangential} = \frac{\pi .d.\omega }{6}$$

where *d* is the rotor diameter in m and $\omega$ is the rotational speed in rpm.

## Hydrodynamic cavitation applied to lignocellulosic biomass pretreatment

**Fig. 2:**
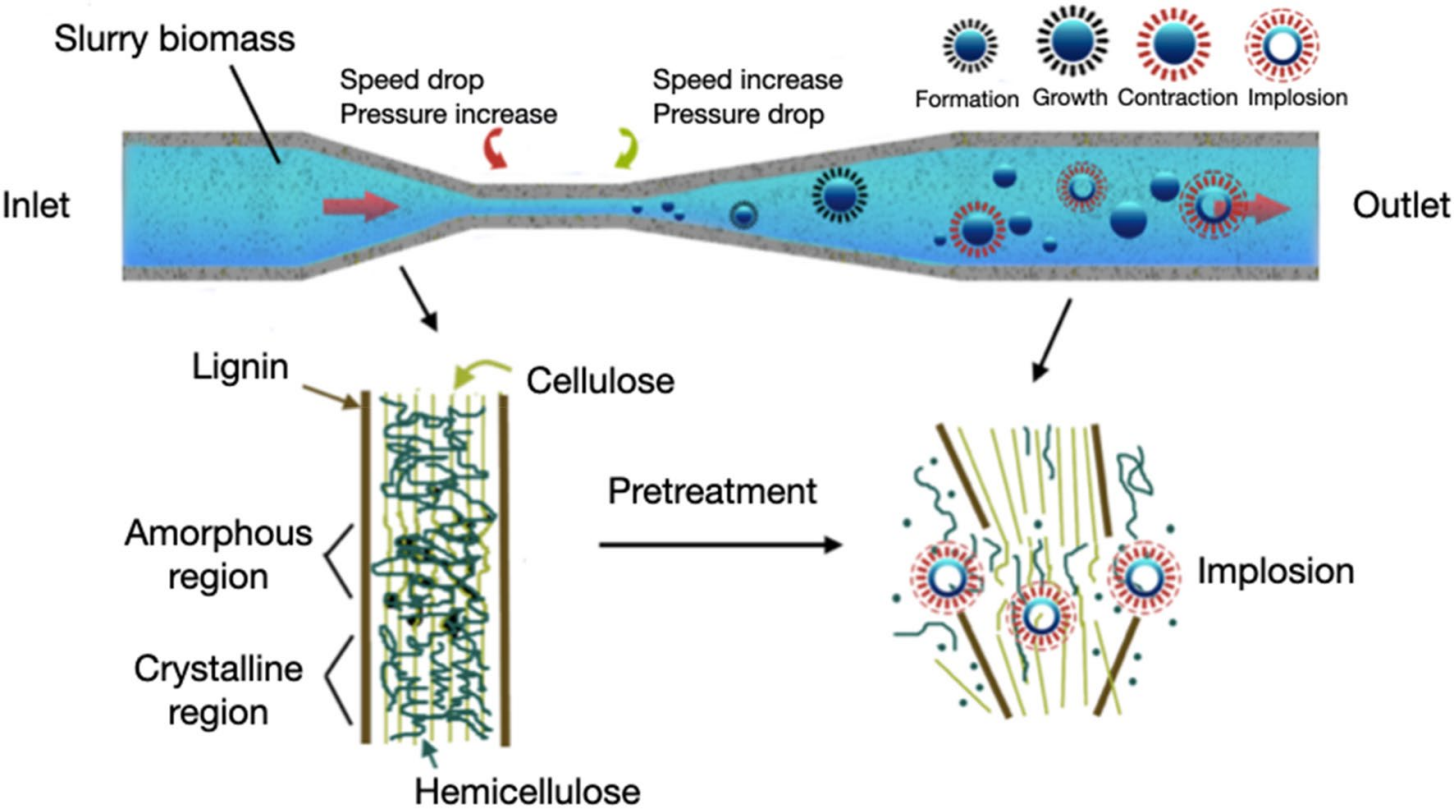
Mechanical effect of hydrodynamic cavitation on lignocellulosic biomass pretreatment

The effects of hydrodynamic cavitation can enhance the pretreatment of lignocellulosic biomass and contribute to delignification and subsequent hydrolysis of carbohydrates. Figure 2 illustrates the lignocellulosic biomass pretreatment via the hydrodynamic cavitation approach.

A recent literature review of hydrodynamic application as lignocellulosic biomass pretreatment is presented in Table 1 highlighting the process parameters, reactor type and the main results obtained in each study.

**Table 1:** - Overview of recent literature illustration on the use of hydrodynamic cavitation reactors to pretreat the various lignocellulosic biomass feedstocks

Raw material (particle size)	Cavitation device	Biomass Placement	Processing parameters	Main results	References
Reed (10 mm)	Orifice plate (27 holes of 1 mm of diameter	Cavitation zone (woven wire cloth 40 mesh)	3% NaOH, 11.8% solid loading, 41.1 min, operating temperature 77 °C, inlet pressure 500 kPa	Lignin removal 35–42%, sugar released 326.5 g/kg of raw reed, 85% of cellulose hydrolised in 72 h. Energy consumption 3.65 MJ/kg of reed	Kim et al. 2015
Sugarcane bagasse (1.18–1.70 mm)	Orifice plate (27 holes of 1 mm of diameter	Cavitation zone (cylindrical wire cloth 40 mesh)	0.48 M NaOH, 4.27% solid loading, 44.48 min, operating temperature 64 °C, inlet pressure 300 kPa	Lignin removal 60.4%, glucan content 52.1%, 97.2% of cellulose hydrolised in 48 h	Hilares et al. 2016
Corn stover (0.25 mm)	Venturi tube (1.8 mm of throat diameter, length 40 mm)	Biomass slurry (Mixed with catalyst and circulated in a closed loop)	0.4 mol/L of Na_2_CO_3_ + 0.6 mol/L of H_2_O_2_, 4% solid loading, 60 min, operating temperature 30 °C, inlet pressure 220 kPa	Lignin removal was verified by FTIR analysis by reduction in the band 1745 and 1606 cm^−1^. Pretreatment efficiency 2.24.10^–5^ g glucose/J	Nakashima et al. 2016
Sugarcane bagasse (10 mesh)	Venturi tube (7.14 mm of throat diameter, length 225.5 mm—15% opening)	Biomass slurry (Mixed with catalyst and circulated in a closed loop)	Ca(OH)_2_ 0.1 g/g of dry biomass, 1% solid loading, 120 min, operation temperature 22 °C, inlet pressure 446 kPa	Changes in composition were measured by crystallinity index. 46% of cellulose hydrolised in 72 h. Sugar released 325 g/kg of raw biomass. Energy consumption 12.8 kJ/g sugar	Madison et al. 2017
Sugarcane bagasse (4.7 mm)	Orifice plate (16 e 27 holes of 1 mm of diameter)	Cavitation zone (cylindrical wire cloth 18 mesh)	0.3 M NaOH, 3 bar, 70 °C, 30 min	Lignin removal 41.83%. 93.05% hydrolysis yield of cellulose and 94.45% hydrolysis yield of hemicellulose in 30 min of pretreatment	Hilares et al. 2017a
Sugarcane bagasse (4.7 mm)	Orifice plate (16 holes of 1 mm of diameter)	Cavitation zone (cylindrical wire cloth 18 mesh)	The alkalis sodium carbonate Na_2_CO_3_ (0.5, 1.0 and 1.5 mol/L), calcium hydroxide Ca(OH)_2_ (0.5 mol/L), potassium hydroxide KOH (0.1, 0.3 and 0.5 mol/L) and sodium hydroxide NaOH (0.3 mol/L), 60 °C, 20 min	In process using NaOH-HC pretreatment: 45.57% lignin removal, 21.98% hemicellulose removal, 62.33% of total carbohydrate fractions were hydrolyzed and 17.26 g/L of ethanol production (0.48 g of ethanol/g of glucose and xylose consumed) was achieved	Hilares et al. 2017b
Corn cob (≤ 212 μm)	Orifice plate (9 holes of 2 mm of diameter	Biomass slurry (Mixed with acetate buffer and circulated in a closed loop)	Laccase enzyme 6.5 U/g of biomass, 5% solid loading, 60 min, operating temperature 30 °C, inlet pressure 50 kPa	Lignin removal 47.4%, cavitational yield 3.56.10^−5^ g/J. Energy consumption 1.35 MJ/kg of biomass	Thangavelu et al. 2018
Cattle Manure and Wheat Straw (2.0 mm)	ARHCR	Worked in a batch mode. Number of cycles per day = 48	Eletric motor 4 kW, 2800 rpm, 35 °C,	Enhanced biogas production to 460 L methane/kg volatile solids. Net energy output 61 kWh/d	Zieliński et al. 2019
Conifer and Eucalyptus pulp	ARHCR	-	6000 rpm, 15.2 L/min, 98 kPa, 9 min, T = 34 °C, 3% solid loading	Paper with tensile index 50.5 kN m/kg, burst index at 3 kPa.m^2^/g. Energy consumption 58.4 kWh/m^3^	Kosel et al. 2019
Sugarcane bagasse (< 0.250 mm)	Venturi tube (1.5 mm of throat diameter, length 40 mm)	Biomass slurry (Mixed with catalyst and circulated in a closed loop)	4.9% NaOH, 2.03% solid loading, 58.33 min, operating temperature 65 °C, inlet pressure 300 kPa	Lignin removal 56.14%, 97.2% enzymatic hydrolysis yield at the optimal condition obtained by RSM	Bimestre et al. 2020
Corncob (≤ 212 μm)	Orifice plate	Biomass slurry (Mixed with acetate buffer and circulated in a closed loop)	Laccase enzyme 6.5 U/g of biomass, 5% solid loading, 60 min, operating temperature 70 °C	Lignin removal 64.1%, Hemicellulose removal 6.57%, saccharification efficiency of 55% with multifunctional cellulases at 50 °C and pH. 5.0	Ganesan et al. 2020
Sugarcane bagasse (10 mesh)	Vortex based cavitation device	Biomass slurry (Mixed with catalyst and circulated in a closed loop)	Solid loading 1% w/v, 1.2m^3^/h, 100 passes at 30 °C, 0.75 M NaOH, 60 min	Lignin removal 77.4%, 75.5% ± 1.9 g/L glucose was released with 70.3% Cellulose hydrolysis, hemicellulose conversion 69.4%	Nalawade et al. 2020
Wheat straw (< 0.05 mm)	ARHCR	Biomass slurry (Mixed with catalyst and circulated in a closed loop)	3000 rpm, 50 °C, 0.4% NaOH, solid–liquid ratio 1:50, 30 min	Lignin removal 24.05%	Lauberte et al. 2021
Miscanthus × giganteus stalks (5 mm)	ARHCR	Biomass slurry (Mixed with catalyst and circulated in a closed loop)	0.3 M KOH, 2200 rpm, 20 min, 7% solid loading, Room Temperature	Lignin removal 41.54%. Paper with Tensile index 24.88 N m/g, burst index at 0.92 kPa m^2^/g	Tsalagkas et al., 2021

The first studies on hydrodynamic cavitation applied to lignocellulosic biomass pretreatment date from 2012 and were conducted by Baxi and Pandit (2012). In this study, hydrodynamic cavitation was used for the delignification of wood. The sawdust was treated with hydrodynamic cavitation (Venturi tube reactor) and alkaline sodium hydroxide solution (5%w/w). The rates of delignification obtained using hydrodynamic cavitation were about 4–5 orders of magnitude greater than those obtained using acoustic cavitation (rate constants for delignification were 9.78 × 10^–6^ and 6.8 × 10^–1^/min for acoustic and hydrodynamic cavitation, respectively).

A hydrodynamic cavitation is a versatile form of pretreatment that can be combined with other methods. Using the synergistic benefits of combined methods has been a new integrated pretreatment approach.

Badve et al. (2014) use hydrodynamic cavitation for intensification of the delignification of wheat straw as an essential step in the paper manufacturing process. Wheat straw was first treated with potassium hydroxide (KOH) for 48 h and subsequently, alkali-treated wheat straw was subjected to hydrodynamic cavitation in an ARHCR. It has been observed that treatment of alkali-treated wheat straw in hydrodynamic cavitation reactor for 10–15 min increases the tensile index of the synthesized paper sheets to about 50–55%, which is sufficient for paper board manufacture.

Kim et al. (2015) studied hydrodynamic cavitation-assisted alkali pretreatment of reed. The cavitation device employed was an orifice plate with 27 holes of a 1 mm diameter. Reactional volume used was 150 mL, with an inlet pressure of 500 kPa at a work temperature of 77 °C. The optimal pretreatment condition was determined as 3% NaOH, 11.8% solid load, and reaction time of 41.1 min, with lignin removal of 35–42% and maximum glucose yield of 326.5 g/kg of biomass after 72 h of enzymatic hydrolysis. The hydrodynamic cavitation as biomass pretreatment also proved to be benefit from an energetic viewpoint with energetic consumption of 3.65 MJ/kg of biomass, a significatively lower value compared with ultrasonic cavitation with a consumption of 14.4 MJ/kg of biomass performed under similar conditions.

Zielinski et al. (2019) monitored the small-scale agricultural biogas plant for biogas production from agricultural residues over a period of 330 days. As the feedstock contained lignocellulosic biomass, ultrasonic pretreatment, and hydrodynamic cavitation pretreatment were used. The final net energy output of agricultural biogas plant (ABP), ABP-Ultrasonic pretreatment, and ABP-Hydrodynamic cavitation pretreatment was, respectively, 56, 52, and 61 kWh/day. Also, in previous works based only on electricity consumption, hydrodynamic cavitation pretreatment of lignocellulosic biomass proved to be more competitive than a microwave (Wu et al. 2019), ultraviolet (Rajoriya et al. 2019), and electric field (Jung et al. 2014).

The lignocellulosic biomass processing steps require operations to be carried out more efficiently from the point of view of energy consumption, processing and production time, the quality of the material produced, and through environmentally friendly processes.

In this sense, Hilares et al. (2016) used hydrodynamic cavitation to optimize the sugarcane bagasse alkaline pretreatment. Under optimized conditions (0.48 M NaOH, 4.27% solid loading, and 44.48 min.) reported 52.1% of glucan content, 60.4% of lignin removal, and 97.2% enzymatic digestibility after 48 h of hydrolysis. Furthermore, the enzymatic hydrolysis of pretreated sugarcane bagasse presents a yield of 82% higher in relation to the bagasse hydrolysis without pretreatment and 30% higher than pretreated bagasse with only alkali. These results suggest that biomass digestibility does not depend only on changes in the composition of the pretreated biomass but has a strong correlation with structural changes that can be attributed to increased biomass porosity due to the mechanical effects of cavitation, favoring the diffusion of enzymes in the substrate (Nakashima et al. 2016). In another study, Hilares et al. (2017a) evaluate the hydrodynamic cavitation using the surface response methodology varying parameters as inlet pressure (1–3 bar), temperature (40–70 °C), and NaOH concentration (0.1–0.3 M). Under optimal conditions (3 bar, 70 °C, and 0.3 M NaOH) were obtained, respectively, 93.05% and 94.45% of hydrolysis cellulose and hemicellulose yield in 30 min of pretreatment. The authors also conducted two new experiments, to optimize the results, achieving a significant reduction in the pretreatment time to 20 min. with an alkali (NaOH) load of 0.3 mol/L in the first study (Hilares et al. 2017b) and reducing the amount of 0.29 M NaOH with the addition of 0.78% v/v H_2_0_2_ in the second study (Hilares et al. 2018), managing to reduce the pretreatment time to 10 min.

To reduce the alkali consumption, the black liquor obtained after first HC pretreatment can be further reused in each additional batch with fresh lignocellulosic biomass. Hilares et al. (2017b) reported that about 80% and 70% of cellulose and hemicellulose hydrolysis yields were achieved in SCB pretreated with black liquor in nine successive repeated batches. This is an important result in terms of favoring the economic viability of pretreatment of lignocellulosic biomass, reducing solvent consumption.

In addition to this approach, the use of biocatalysts to remove recalcitrant fractions from lignocellulosic biomass can also be combined with hydrodynamic cavitation. Thangavelu et al. (2018) combined the hydrodynamic cavitation using an orifice plate with the enzymatic pretreatment for corncob to biofuel production. The most significatively parameters of the study were enzyme load (3–10 U g^1^), solid load (2.5–5%), and reaction time (5–60 min). On optimized conditions (6.5 U g^1^, 5%, and 60 min) obtained 47.4% of lignin removal, cavitation yield of 3.56.10^–5^ g/J, and energy consumption calculated at 1.35 MJ/kg.

Considering the potential of hydrodynamic cavitation, different operating modes have been studied, such as semi-continuous and continuous, both interesting alternatives from the operational point of view for biorefineries and other industrial applications. These processes save time, energy, and operating costs compared to the batch process if we consider that the biomass does not recirculate in the reactor, being treated only once, ensuring greater productivity (Lee et al. 2015; Hilares et al. 2019). Some research on continuous pretreatment methods with potential large-scale applications has been reported by Chen et al. (2013), Choi and Oh (2012), Vandenbossche et al. (2016), and Han et al. (2013).

## Influencing parameters on hydrodynamic cavitation intensity to lignocellulosic biomass pretreatment

Is essential the identification of vital process parameters and the comprehension of the influence of these parameters involved in physical and chemical mechanisms (Gogate and Patil 2015). The main influence parameters in a hydrodynamic cavitation reactor to lignocellulosic biomass pretreatment can be classified in three groups:

### Reactor structural characteristic (geometry of cavitation device)

In cavitation devices with Venturi pipe, a narrower throat causes a greater pressure gradient and consequently stronger cavitation, resulting in greater pretreatment efficacy compared to a case with a wider throat. Nakashima et al. (2016) assessed the throat diameter Venturi pipe influence in corn stover pretreatment. In this study, the greater release of glucose of 4 g/L in enzymatic hydrolysis was reached when the 1.4 mm throat diameter was employed with a cavitation number of 0.29 compared with the case of 1.8 mm throat diameter with cavitation number equal to 0.44 and glucose release of 3 g/L. The ratio of length and throat diameter (*l*/*d*) is a parameter that plays an important role in cavities growth control and in its final collapse conditions, concluding that the increase in *l*/*d* ratio slows down the pressure recovery on Venturi pipe (Simpson and Ranade 2019). In cavitation reactors of the Venturi type, the recovery pressure rate is controlled by its divergence angle. This angle should vary from 5.5° to 7.5° for a Venturi pipe applied to hydrodynamic cavitation, where the recovery pressure rate must be favorable. High divergence angle values lead to sudden expansion and hydraulic losses raise. The expansion induces a loss due to the flow separation on walls and a secondary turbulent flux in the tube divergent section (Ashrafizadeh and Ghassemi 2015).

In orifice plates, the number and diameter of the orifices are fundamental parameters also. An area increase of orifices can raise the cavitation number and consequently reduce the expected effects. Hilares et al. (2017a) verified the cavitation number reduction using orifice plates with 16 holes of 1 mm ($\sigma_{c}$ = 0.017) besides the high efficiency in sugarcane bagasse pretreatment in relation to plates with 27 holes of 1 mm ($\sigma_{c}$ = 0.048).

These results are in accordance with the proposal that sugarcane bagasse pretreatment presents high efficiency with a low cavitation number. In systems with orifice plates, the $\alpha$ and $\beta$ parameters need to be calculated (Sivakumar and Pandit 2002) is given as:

10 $$\alpha = \frac{{\text{total perimeter of the holes}}}{{\text{total area of opened}}} = \frac{4}{{d_{0} }} \left[ {{\text{mm}}^{ - 1} } \right]$$

11 $$\beta = \frac{{{\text{Sum of the hole area}}\left( {\text{s}} \right){\text{on the orifice plate}}}}{{\text{cross sectional area of the pipe}}} = n\left( {\frac{{d_{0} }}{D}} \right)^{2}$$

where $d_{0}$ is the orifice diameter, $D$ is the pipe diameter and $n$ is the number of holes in the orifice plate. The $\beta$ value significatively influences the cavitation number. Lower values of $\beta$ result in lower cavitation numbers. Already the value of $\alpha$ depends on the number of orifices in the plate and its diameter. Hilares et al. (2020) observed that the reduction sugars released in pretreated sugarcane bagasse enzymatic hydrolysis were a linear function of geometrical parameters $\alpha$ (directly proportional) and $\beta$ (inversely proportional). Furthermore, smaller orifice diameters allow high-velocity flow, high-frequency turbulence, and greater shear area, which results in greater collapse pressure, eliminating posterior resistance to mass transfer moreover leading to higher process efficiency (Ghayal et al. 2013; Chuah et al. 2016).

For ARHCRs the intensity of cavitation depends on the rotation speed. Higher rotation speeds lead to a lower cavitation number and a consequent increase in cavitation intensity. With higher rotation speeds, turbulence intensity increases, thus increasing the intensity of the bubble collapse (Kosel et al. 2019; Petkovšek et al. 2015).

### Liquid media and biomass features

To reach efficient hydrolysis, the use of synergic benefits of combined methods may be a new integrated lignocellulosic biomass pretreatment approach. Chemical products as sodium hydroxide (NaOH) and lime [Ca(OH)_2_] are being used in combined pretreatment of hydrodynamic cavitation of sugarcane bagasse and reed (Kim et al. 2015; Madison et al. 2017) and sodium percarbonate (2Na_2_CO_3_ e 3H_2_O_2_) in corn stover pretreatment (Nakashima et al. 2016). The use of mechanical strengths as hydrodynamic cavitation increases the chemical reagents efficiency in pretreatment with excellent effectiveness in lignocellulosic biomass delignification, silica removal, partial hemicellulose removal, and cellulose swelling, resulting in a substantial increase in fiber superficial area, porosity, and cristanility changes (Carvalho et al. 2016). The biomass particle size can also affect the pretreatment efficiency in hydrodynamic cavitation systems as the heat and mass transfer. A particle size reduction increases the exposed biomass superficial area, beyond favoring the heat and mass transfer resulting in higher total conversions (Khullar et al. 2013). In continuous pretreatment systems, where the powdered biomass is mixing with the catalyst forming a biomass slurry, which is pumping in a closed loop. In these systems, the particle size must be observed so that it does not occur clogging in the pump and cavitation device. An alternative solution to maintaining the biomass isolated in the cavitation zone avoiding the solid particle circulation on the orifices (Hilares et al. 2017a).

### Technological process features

The liquid temperature is an important parameter, which can affect the cavitation efficiency. Vapor fluid pressure depends on temperature and increases exponentially with it. Besides, the solution properties as density, viscosity, and surface tension are affected too. That way increases the fluid temperature will result in a proportional increase in reaction velocity and cavitation effects. However, after reaching very high temperatures (70 °C) the effects decrease (Šarc et al. 2018; Hilares et al. 2017a). As the effects of temperature on cavitation intensity for pretreatment of lignocellulosic biomass are contradictory at high temperatures, a compromise value must be obtained by optimization in real applications (Sun et al. 2021a).

Inlet pressure is an important parameter also because the developed vapor cavities depend on it. A pressure increase tends to turn vapor bubble collapse more violent, liberating a high number of radicals OH and reducing the cavitation number (Gogate and Patil 2015). Meanwhile, very high pressures on the inlet can cause the supercavitation phenomena which is a vapor cavities cloud formation whose collapse is significatively dampened leading to a decreasing cavitational effectivity. In general, inlet pressure values on 3–5 bar range have been considered ideal to Venturi pipe and orifice plates set up to hydrodynamic cavitation conditions applied to lignocellulosic biomass (Sharma et al. 2019; Hilares et al. 2016).

The duration of pretreatment is also an important parameter. Usually, longer durations are better for process efficiency. However, long times considerably increase the pretreatment costs and possibly generate more by-products for example inhibitors such as 5-hydroxymethylfurfural, furfural, phenolic acids, and aromatic compounds (Zhao et al. 2021; Parawira and Tekere 2011).

As observed are many factors that affect the performance and design of cavitation reactors. The optimization of these parameters can be done from the design of experiments (DOE), which is a statistical approach that allows the variation of different parameters at the same time. The most used method in the pretreatment of lignocellulosic biomass is the response surface methodology (RSM) whose objective is to optimize a response influenced by several factors (Nalawade et al. 2020; Bimestre et al. 2020; Hilares et al. 2020).

Due to the flow complexity and necessary measurement instrumentation to make experimental flow characterization, allied to complex geometry and low temporal scales the computational fluid dynamics can be an important tool in the cavitation reactors design (Abbas-Shiroodi et al. 2021).

## Hydrodynamic cavitation at industrial level

Adaptation of hydrodynamic cavitation pretreatment to industrial level requires a lignocellulosic biomass sustainable conversion with costs minimization and process efficiency maximization. Hydrodynamic cavitation-based technology appears as a promising biomass pretreatment at industrial scales, which may be tailored to continuous or semi-continuous operation saving time, energy, and operational costs. The fact that hydrodynamic cavitation reactors have a simple setup, and are easily scalable if compared to other cavitation reactor options, can be installed in any existing production process as a separate module without major process modification (Sun et al. 2021a; Hirooka et al. 2009). Both combined methods and recycled chemical products usage aggregate environmental and sustainable economics to the process (Verdini et al. 2021).

Ramirez-Cadavid et al. (2014) used cavitation in a commercial-scale corn ethanol production process to release and hydrolyze unconverted carbohydrate fractions. The results show that cavitation altered the particle size distribution led to qualitative changes in cell structure, increased the total sugars after liquefaction, reduced the total solids after liquefaction, and led to significant increases in ethanol production and solids conversion during SSF. Simple energy and economic analysis showed that the energy return of cavitation in the form of ethanol is 16 times greater than the energy expended to generate the cavitation. Furthermore, the value of the extra ethanol produced by cavitation was 38 times more than the cost of the electricity used for the cavitation system.

Garuti et al. (2018) in a full-scale application of a hydrodynamic cavitation system in an agricultural biogas plant investigated the unique pretreatment without recirculation for enhancing the methane potential from agricultural biomasses with molasses and cornmeal as supplementary energy sources. In 6 months of operational data showed that pretreatment with hydrodynamic cavitation maximized specific methane production by about 10%. Furthermore, hydrodynamic cavitation affected the viscosity and particle size of digestate, contributing to reduced energy demand for mixing, heating, and pumping.

## Future perspectives

Hydrodynamic cavitation is an extremely complex phenomenon and, as such, its application in the pretreatment of lignocellulosic biomass has some limitations and challenges that should be addressed in future research.

It is essential to identify the vital parameters of the process and understand the influence of these parameters on the physical and chemical mechanisms involved, as well as their combined effects. The optimization of these parameters is extremely important for the method's viability. Transport properties of the reaction mixture, the heat and mass transfer of the system, the chemical kinetics of the reaction medium (Badve et al. 2015) in addition to the complexity and variability of the proportions of chemical structures in biomass that impact its biodegradability require further research and investigations.

The hydrodynamic cavitation-assisted biomass pretreatment process has some disadvantages related to the use of different chemicals and biomass recovery process. Furthermore, it uses large volumes of water to reach the required pH of the biomass for use in the subsequent steps of enzymatic hydrolysis and fermentation (Prado et al. 2021). Due to the strong collapse of cavitation bubbles, erosion and vibration problems in reactors can affect their durability and performance, requiring further investigations in this regard (Sun et al. 2021a).

Future investigations for hydrodynamic cavitation should be conducted with hydrogen peroxide (Valim et al. 2017) and ozone (Osório-González et al. 2020) beyond the Fenton reactions (Fenton reaction and Fenton-like reaction) releasing hydroxyl radicals (OH) and hydroperoxyl (HO_2_) oxidizing and degrading the recalcitrant structures of lignocellulosic biomass making easier the access of enzymes to cellulose and enhancing the enzymatic hydrolysis (Liu et al. 2020). An important aspect to be considered is the development of recovery and reuse strategies for the chemicals used, such as the coupling of alkali recovery membranes, as well as to valorize lignin–hemicellulose fractions or derivatives from the wastewater (Prado et al. 2021).

In addition, CFD computational fluid dynamics can be used from the conceptual phase of reactor design, helping to determine the best product solution until the production stage, allowing to represent of different scenarios. The use of advanced experiments such as high-speed photograph and particle image velocimetry should also be encouraged (Sun et al. 2021a).

More accurate studies of economic feasibility analysis with a survey of operating costs, maintenance, and investments in equipment are necessary.

## Conclusions

This review highlighted the importance and hydrodynamic cavitation potential as an alternative to conventional methods of lignocellulosic biomass pretreatment. The reactor setup has a fundamental importance in the process as well as the operational conditions as temperature, inlet pressure, cavitation number, and particle size turning the process economically viable in the biorefinery context. Hydrodynamic cavitation has shown an excellent alternative to large-scale processing, friendly ecological, energetically efficient, and can be employed together with other pretreatment ways. Future investigations should concentrate on high-performance reactor design with low costs and combined pretreatment methods.

## Data Availability

The data supporting the conclusions are included in the main manuscript.
